# Recollections

**Published:** 1920-05-08

**Authors:** George Makins


### RECOLLECTIONS.

### By Sir George Makins, G.C.M.G., P.R.C.S.

The early period of Burdett's career must have been one of the most strenuous of the whole; he was attempting to keep his terms at Guy's Hospital in order to fulfil his ambition to become a qualified practitioner, he was preparing his book on cottage hospitals (which did much to help on a movement then in its infancy), and he was cultivating his journalistic instincts and writing on many subjects, particularly on those relating to public health. Much work was also done at this period which was to lead up to his great book on the hospitals and asylums of the world. Then, to the surprise of all his friends, he suddenly plunged into the business life of the City and into the realms of public finance.

The writer is unable to follow Burdett into the years spent in the City. It was characteristic of the man and his boundless energy and self-confidence that he should suddenly cut himself entirely adrift from an assured position which he had himself built up to enter on a fresh enterprise which would bring him into intimate association, and often into opposition, with the keenest minds in the business world. Suffice it to say that he brought with him business capacity gained in his earlier field, a knowledge of men and human nature, and powers of administration which rapidly permitted him to make his mark and prove a power that had to be reckoned with. What was of more importance to the outside world was that the new work seemed to stimulate his zest for the old, and, moreover, it brought him into connection with men of means whom he found ready and willing to help forward the many schemes for the public good which he took in hand.

If but mere mention is made of some of these schemes—the Home Hospital for Paying Patients in Fitzroy Square, the Nurses' Co-operation, the Nurses' Pension Fund, active co-operation with the managers of the Hospital Sunday Fund, King Edward's Hospital Fund for London, the League of Mercy, parochial organisation in the parish in which he lived, short excursions into the politics of the day, and many literary ventures—the magnitude and scope of his interests and the success which attended his efforts is astounding. Any one of these schemes successfully launched would have sufficed in itself to establish a reputation for its promulgator, and the solidity with which they have taken root speaks more forcibly in this sense than can any words of the writer of an obituary note.

The multiplicity and importance of these undertakings illustrate a very striking feature in Burdett's career—the faculty which he possessed of turning his attention in a fresh direction when he felt that a new fledgeling was firmly enough established to walk alone. Not that he lost interest, for he was always ready and at hand if difficulties should arise, but he had the feeling that his part of the work was done for the time, and that his activities might more profitably be utilised in other spheres.

Engrossing as his many duties were, Burdett found time and opportunity to travel widely on the Continent and in America, carrying with him new ideas and bringing back with him experience capable of utilisation in his own country. Thus he became widely known on both sides of the Atlantic, and his advice and experience were asked for from many quarters.

Burdett's latest essay as an editor and journalist was a natural sequence to the continuous activity he had shown as a contributor to the Press. As a leader writer and the author of letters in the daily Press he had done much to influence public opinion on many subjects, and the devotion of the last years of his life to his own paper furnished a suitable and happy occupation for him.

His personal character is a difficult one to analyse. Generous and kind-hearted, capable of maintaining an astonishing control of his temper, yet he was of an essentially combative disposition. He was never more happy than when striving with the difficulties of a new undertaking or when defending himself from the criticisms of others. It must be remembered that Burdett's method of defence consisted in taking the offensive. He hit out strongly, whether in words or with the pen; thus he made many enemies, and his tactics not unfrequently did not commend themselves to his opponents. Yet he himself took such blows as he received courageously, soon let them pass out of remembrance, and bore no malice. Here, indeed, was one of the main sources of his strength and an explanation of his capacity to persevere with the matter in hand when many men would have retired from the contest with disappointment or disgust. His industry was astonishing, although his method of work into the early hours of the morning was not always congenial to his coadjutors. Nevertheless, he gained the respect and confidence of those who worked with him, and a connection with him served as a passport for many to positions into which his influence allowed him to help them.

In spite of the strenuous and busy life he lived, Burdett found time for relaxation and amusement. In his younger days he was an enthusiastic tennis player, he was part owner of a motor-boat (of which the writer has pleasant reminiscences), he was fond of billiards, and in his latter days took to croquet.

He delighted in the exercise of hospitality, and many were the kind acts he performed for those in trouble or distress. His death is not only a public loss, but he will be missed by a large circle of friends whose private association with him allowed them to form a just estimate of his many-sided nature.

